# Early‐Onset Medullocervical Low‐Grade Glioma With FGFR1 Mutation and Leptomeningeal Spread in an Infant: A Case Report

**DOI:** 10.1002/ccr3.72974

**Published:** 2026-06-18

**Authors:** Thaer A. Tumar, Mahdi W. Suboh, Mones H. Atatre, Hanin Shatrit, Qais M. Alnjoom

**Affiliations:** ^1^ Department of Clinical Medical Sciences, Faculty of Medicine and Health Sciences Palestine Polytechnic University Hebron Palestine; ^2^ Department of Radiology Al‐Ahli Hospital Hebron Palestine

**Keywords:** FGFR1 mutation, hydrocephalus, leptomeningeal dissemination, medullocervical tumor, pediatric low‐grade glioma

## Abstract

Pediatric low‐grade gliomas (PLGGs) are generally slow‐growing tumors associated with favorable long‐term outcomes. However, their occurrence in early infancy is rare, particularly when arising in the posterior fossa with extensive dissemination and hydrocephalus. Advances in molecular profiling have identified specific genetic subtypes, including FGFR1‐mutated gliomas, which may demonstrate more aggressive clinical behavior than suggested by histology alone. We report a four‐month‐old female who presented with signs of increased intracranial pressure and sunsetting eyes. Imaging revealed a heterogeneously enhancing exophytic medullary mass with a large cystic component causing tetraventricular hydrocephalus. Following ventriculoperitoneal shunt placement and surgical decompression, histology confirmed a low‐grade glioneuronal tumor with low proliferative activity. Molecular analysis identified an FGFR1 mutation and 18q13 deletion, and methylation profiling classified the tumor within the MYB(L1)‐family subtype B. Despite benign histologic features, the tumor progressed with cervical cord extension and diffuse spinal leptomeningeal metastases. Targeted therapy with trametinib achieved partial radiologic response before further progression. The patient remains clinically stable under ongoing therapy and multidisciplinary care. This case underscores the critical role of molecular diagnostics in risk stratification and treatment selection, particularly in infants with atypically aggressive PLGG.

## Key Clinical Message

Infantile low‐grade gliomas can exhibit aggressive clinical behavior despite benign histology and low proliferative indices. Early integration of comprehensive molecular profiling—particularly identification of FGFR1 alterations—is essential for accurate risk stratification and guiding targeted therapy in atypical pediatric cases with dissemination and rapid progression.

## 1 Introduction

Gliomas are neuroepithelial tumors that arise from the supporting glial cells of the central nervous system (CNS). They include astrocytomas, oligodendrogliomas, mixed oligoastrocytic tumors, and mixed glioneuronal tumors, which originate from astrocytic, oligodendroglial, mixed oligoastrocytic, or neuronal–glial cells, respectively [1]. According to the World Health Organization (WHO) classification of CNS tumors, gliomas are graded from 1 to 4 based on histopathological and molecular characteristics, with lower‐grade tumors generally demonstrating slower growth and more favorable outcomes than higher‐grade tumors [2].

Low‐grade gliomas (LGGs), which correspond to WHO grade 1 and grade 2 CNS tumors, are the most common type of brain tumor in children, accounting for approximately 30%–40% of all pediatric CNS tumors. The annual incidence rate in the United States is estimated at 2.1 per 100,000 children [3]. Grade 1 gliomas, such as pilocytic astrocytomas and subependymomas, are typically well circumscribed, slow‐growing, and may be curable with complete surgical resection. Grade 2 gliomas, including diffuse astrocytomas and oligodendrogliomas, are generally more infiltrative but remain relatively indolent compared with higher‐grade tumors. In contrast, high‐grade gliomas (WHO grades 3 and 4) are characterized by increased mitotic activity, cytologic atypia, microvascular proliferation, and/or necrosis, leading to more aggressive clinical behavior [1]. Grade 3 gliomas, such as anaplastic astrocytomas, anaplastic oligodendrogliomas, and anaplastic ependymomas, typically demonstrate an intermediate clinical course, whereas grade 4 CNS tumors are associated with rapid progression and poor prognosis [4].

Diffuse low‐grade gliomas in children commonly present with variable neurological manifestations depending on tumor location, including seizures, focal neurological deficits, headaches, vomiting, visual disturbances, and gait abnormalities [5]. Hydrocephalus is an uncommon but clinically significant initial presentation, particularly in tumors involving the brainstem or posterior fossa.

Fibroblast growth factor receptors (FGFRs) are transmembrane tyrosine kinase receptors involved in cellular proliferation, differentiation, migration, and survival. Recent genomic studies have identified recurrent FGFR1 mutations in pediatric gliomas, particularly in pilocytic astrocytomas and other low‐grade subtypes. The most frequently reported alterations involve codons Asn546 and Lys656 and are considered oncogenic hotspot mutations. Tumors harboring FGFR1 mutations, similar to the mutation identified in our patient, have been associated with more aggressive biological behavior and less favorable clinical outcomes compared with tumors lacking these alterations. These findings support the growing role of molecular profiling and targeted therapeutic strategies in selected pediatric LGGs [6].

Magnetic resonance imaging (MRI) remains the gold standard for the initial diagnosis and evaluation of low‐grade gliomas [7]. In addition, emerging neuroradiological approaches based on nuclear medicine techniques are increasingly being utilized for improved tumor characterization and longitudinal follow‐up [8]. These tumors characteristically appear as nonenhancing lesions on CT or MRI. Management is primarily based on surgical intervention, although complete resection is not always feasible because of tumor location or infiltrative behavior. Adjuvant therapy may include chemotherapy and/or radiotherapy depending on tumor progression and clinical presentation [9]. Overall outcomes in pediatric LGGs are generally favorable, with population‐based studies reporting an estimated overall survival of approximately 87% [10]. However, cases such as ours, involving an FGFR1‐mutated medullocervical tumor with early hydrocephalus, spinal progression, and leptomeningeal spread, underscore the biological heterogeneity of these tumors and the importance of individualized, molecularly guided treatment strategies.

## 2 Case History / Examination

A four‐month‐old full‐term female infant, born after an uneventful pregnancy and delivery, presented with a one‐day history of hypoactivity and abnormal eye positioning. Her mother described a persistent “staring look,” with downward and forward deviation of the eyes. On examination, the anterior fontanelle was open and bulging, and the infant demonstrated the classic “sunsetting” eye sign, suggestive of increased intracranial pressure. Vital signs were stable, and no focal neurological deficits were identified at the time of presentation.

## 3 Methods (Differential Diagnosis, Investigations, and Treatment)

Given the clinical signs of raised intracranial pressure and posterior fossa involvement, differential diagnoses included brainstem glioma (low‐grade versus high‐grade), ependymoma, medulloblastoma, atypical teratoid/rhabdoid tumor (AT/RT), and other embryonal posterior fossa tumors. Initial transfontanelle ultrasonography revealed marked ventricular dilatation consistent with hydrocephalus. Subsequent brain MRI demonstrated a heterogeneously enhancing exophytic mass arising from the right posterolateral medulla oblongata, with a prominent cystic component and massive tetraventricular hydrocephalus (as shown in Figure 1).

**FIGURE 1:**
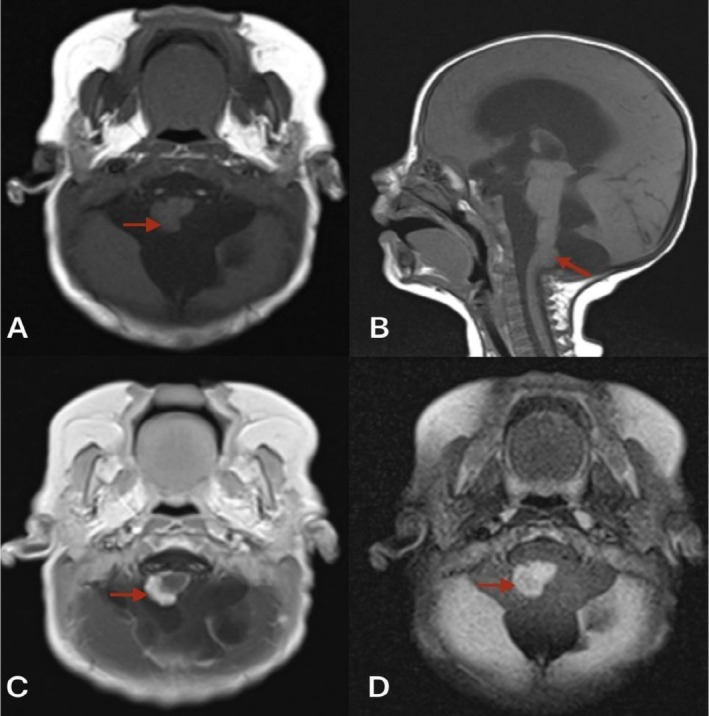
Shows an exophytic mass (red arrow) arising from the right posterolateral medulla oblongata, measuring approximately 1.3 × 1.1 × 1.6 cm (anteroposterior × transverse × craniocaudal). The lesion is isointense to the adjacent medulla on T1‐weighted axial (A) and sagittal (B) images, heterogeneous with focal hyperintense areas on T2 (D), and demonstrates minimal diffusion restriction on DWI and ADC sequences. Post‐contrast T1 images (C) show homogeneous vivid enhancement, while SWI reveals minimal blooming artifact suggestive of a small hemorrhagic component. Posterior to the mass, a cystic lesion measuring approximately 2.4 × 2.6 × 2.3 cm is present, resulting in marked secondary tetraventricular hydrocephalus involving the lateral, third, and fourth ventricles (best seen in B).

A ventriculoperitoneal (VP) shunt was placed to relieve hydrocephalus. During the first year of life, follow‐up imaging demonstrated progressive tumor enlargement with extension into the upper cervical spinal cord. Comprehensive MRI of the brain and spine revealed significant craniocaudal progression measuring up to 4.0 cm, with involvement of the lower midbrain, cerebellar vermis and tonsils, and extension down to the C4–C5 spinal level (as shown in Figure 2). The VP shunt remained functional with normalization of ventricular size. The patient subsequently underwent posterior fossa decompression with C1 laminectomy and tumor biopsy. Histopathological examination confirmed a low‐grade glioneuronal neoplasm, with positive GFAP and synaptophysin immunostaining and a low Ki‐67 proliferation index. Despite these benign histological features, the tumor demonstrated aggressive clinical behavior. Molecular analysis revealed an FGFR1 mutation and deletion of chromosome 18q13. Methylation profiling classified the tumor as a MYB(L1)‐family low‐grade glioma, subtype B.

**FIGURE 2:**
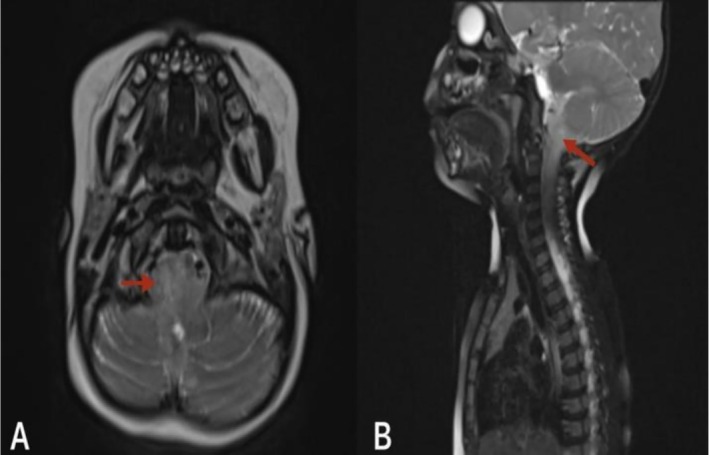
Follow‐up brain MR images and T2‐weighted images. Axial (A) and sagittal (B) planes show worsening of the dorsolateral exophytic medullary mass lesion (red arrow), which is larger in the current examination and extends down to the upper cervical spinal cord, with diffuse spinal drop metastasis as well as dural and leptomeningeal metastasis. Adequate decompression of the ventricular system via a right‐sided VP shunt was noted (no hydrocephalus).

### 3.1 Treatment

Following surgical decompression, the patient recovered well and was discharged in stable condition. However, serial imaging demonstrated continued tumor progression. In light of the identified FGFR1 mutation and clinical deterioration, targeted therapy with the MEK inhibitor trametinib was initiated at a dose of 0.45 mg orally once daily. Treatment response was monitored using tumor fluid content volume (TFCV), which decreased from 9.9 at treatment initiation to 6.0 after 1 year and further to 0.45 at subsequent follow‐up, indicating a partial radiological response.

## 4 Conclusion and Result (Outcome and Follow‐Up)

Despite initial radiological improvement, later imaging revealed renewed tumor progression while on ongoing therapy. At the most recent evaluation in early childhood, the patient remained clinically stable on trametinib under regular multidisciplinary neuro‐oncology follow‐up. Neurological examination revealed no cranial nerve deficits except for a newly suspected right Horner's syndrome. A positive Gower sign indicated proximal lower limb weakness, and intermittent right leg limping was noted, previously responsive to corticosteroids. Mild sleep apnea was also reported. Overall, the patient demonstrated relative clinical stability despite radiological fluctuations, underscoring the discordance between low‐grade histology and aggressive tumor behavior and emphasizing the importance of integrating molecular profiling into prognosis and therapeutic decision‐making.

A summary of the clinical course and management timeline is provided in Table 1.

**TABLE 1:** Summarizes the chronological clinical course, radiological evolution, therapeutic interventions, and outcomes of this infant diagnosed with pediatric low‐grade glioma.

Age/time point	Clinical event	Imaging findings	Intervention	Outcome
4 months (Presentation)	Hypoactivity, bulging fontanelle, sunsetting eyes	MRI: Exophytic medullary mass with large cystic component and tetraventricular hydrocephalus	VP shunt placement	Resolution of hydrocephalus
First year of life	Progressive tumor enlargement	Extension into upper cervical spinal cord (C4–C5)	Posterior fossa decompression + C1 laminectomy + biopsy	Histology: Low‐grade glioneuronal tumor, low Ki‐67
Post‐biopsy molecular analysis	—	FGFR1 mutation, 18q13 deletion; MYB(L1)‐family subtype B	Molecular classification	High‐risk biological profile identified
Follow‐up (progression)	Continued radiologic progression	Cervical extension and leptomeningeal dissemination	Initiation of trametinib (0.45 mg daily)	Partial radiologic response
1 year on trametinib	Clinically stable	TFCV reduced (9.9 → 6.0 → 0.45)	Continued targeted therapy	Partial response maintained
Later follow‐up	Mild neurologic deficits (suspected Horner's syndrome, Gower sign, mild sleep apnea)	Radiologic fluctuation with renewed progression	Ongoing trametinib + multidisciplinary care	Clinically stable despite imaging progression

## 5 Discussion

The most prevalent brain tumors in children are pediatric low‐grade gliomas (PLGGs), which usually have a favorable long‐term prognosis and an indolent course [11, 12]. However, their presentation in infancy, particularly their aggressive behavior and widespread dissemination, is exceedingly rare and poses significant diagnostic and therapeutic challenges [12]. This case study sheds light on this unusual situation and provides insightful information on the unusual clinical manifestations, diagnostic challenges, uncommon genetic traits, and ensuing treatment conundrums that arise while treating disseminated PLGG in a noticeably young child.

The initial clinical presentation at 4 months of age was marked by hypoactivity, a bulging fontanelle, and the sunset eye sign, strongly indicating hydrocephalus. This early onset of symptoms is highly unusual for PLGGs, which more commonly manifest between 5 and 14 years of age [12]. High‐grade, aggressive tumors such as medulloblastomas or atypical teratoid rhabdoid tumors (ATRTs) are usually considered in the differential diagnosis for this age range [13]. The presence of sunsetting eyes, a classic sign of increased intracranial pressure, further underscores the urgency of the clinical situation.

Neuroimaging plays a crucial role in establishing the initial diagnosis and tracking disease progression. Transfontanelle ultrasound identified hydrocephalus, prompting further investigation with MRI [12]. MRI revealed an exophytic tumor with a cystic component in the posterior fossa, initially raising the possibility of anatomical cysts such as Blake's pouch cysts, which can be challenging to differentiate from solid tumors in infants [12]. Subsequent imaging, however, revealed leptomeningeal dissemination to the spinal cord, a critical finding that significantly altered the prognostic outlook. This widespread dissemination, commonly referred to as drop metastases, is a rare phenomenon in PLGGs; it is reported in fewer than 5% of cases and is more typically associated with high‐grade malignancies [14, 15].

Histopathological examination confirmed a low‐grade glioneuronal tumor with positive staining for GFAP and synaptophysin and a remarkably low Ki‐67 proliferation index of less than 1% [15]. This low proliferative activity strongly contrasted with the widespread dissemination observed. Molecular profiling identified an FGFR1 mutation and classified the tumor as a MYB(L1) family subtype B FGFR1 alteration, whereas recognized drivers in PLGG are less common than BRAF V600E mutations or KIAA1549‐BRAF fusions [13, 16]. The combination of a low‐grade histological appearance, a low Ki‐67 index, and widespread dissemination presents a paradox, as aggressive behavior is typically linked to high proliferative rates and specific high‐risk molecular alterations [16]. This unusual discordance between the benign histological features and aggressive clinical course underscores the complexity of PLGG in infants and suggests that uncharacterized genetic or biological factors may drive the propensity for dissemination [14].

The aggressive nature of the disease, despite its low‐grade histology, poses significant therapeutic challenges. Complete surgical resection was not possible because of the tumor's location and diffuse nature of the tumor [12]. The management of hydrocephalus necessitates ventriculoperitoneal shunt placement [12]. Given the identified FGFR1 mutation and the established role of the MAPK/ERK pathway in PLGG pathogenesis, targeted therapy with the MEK inhibitor trametinib was initiated [11, 17]. FGFR1 alterations are known to activate downstream intracellular signaling pathways, particularly the RAS/MAPK/ERK pathway, which contributes to tumor proliferation and survival in pediatric low‐grade gliomas [13, 16]. Trametinib is a selective MEK inhibitor that targets this downstream signaling cascade and suppresses MAPK pathway activation. Therefore, although trametinib does not directly inhibit FGFR1 itself, it represents a form of targeted therapy aimed at blocking the oncogenic signaling pathway activated by the FGFR1 mutation [11, 13]. MEK inhibitors have demonstrated promising results in pediatric gliomas, particularly in patients with MAPK pathway alterations [17].

Other disease‐directed therapeutic options for disseminated pediatric low‐grade glioma include conventional systemic chemotherapy and radiotherapy. Standard chemotherapy regimens, such as carboplatin/vincristine or vinblastine, have historically been used in progressive or unresectable PLGGs, particularly in very young children in whom radiotherapy is preferably delayed because of its significant long‐term neurocognitive and endocrine toxicities [9, 12]. Although radiotherapy may provide local tumor control, its use in infancy remains limited because of the potential risk of neurodevelopmental impairment and other late adverse effects [12]. In the present case, the patient's young age, disseminated disease pattern, and identified FGFR1‐associated MAPK pathway alteration favored the use of targeted therapy with trametinib as a biologically directed treatment strategy [11, 13].

Initially, the patient achieved a partial response to trametinib, with a notable reduction in tumor volume [17]. This initial positive response aligns with the reported efficacy of MEK inhibitors in PLGG [11, 17].

Despite its promising efficacy, trametinib therapy may be associated with several adverse effects requiring close clinical monitoring. Reported toxicities in pediatric patients include skin rash, diarrhea, paronychia, mucositis, fatigue, and elevated creatine phosphokinase levels [11, 17]. Less common but clinically significant adverse events include cardiomyopathy, ocular toxicity, and hypertension, emphasizing the importance of multidisciplinary follow‐up during treatment [17].

However, despite continued treatment, the disease subsequently relapsed, indicating the development of acquired resistance to targeted therapy. Such acquired resistance is a known challenge in targeted therapies for cancer, necessitating further research into the underlying mechanisms and potential combinatorial strategies [11]. Studies have shown that some patients experience relapse or progression after dose reduction or discontinuation of MEK inhibitors, and the long‐term efficacy of monotherapy can be difficult to achieve [17].

In addition to the direct effects and progression of the tumor, the patient experienced serious long‐term neurological aftereffects, such as mild sleep apnea, suspected Horner's syndrome, and muscular weakness (Gower sign). These side effects underscore the vital need for all‐encompassing supportive care, such as seizure control, nutritional support, and physical therapy, to maximize the patient's quality of life during the withdrawn and difficult course of treatment. The persistent difficulties highlight that although vigorous disease control is crucial, tailored care approaches for these complicated cases should prioritize reducing treatment‐related side effects and maintaining long‐term quality of life [12].

This case underscores the extreme rarity of disseminated low‐grade glioma presenting in early infancy with aggressive features despite their benign histology. The unique combination of early onset, widespread dissemination, and rare molecular alterations challenges the current understanding of PLGG biology and calls for further in‐depth molecular characterization of these tumors. Continued research is vital to elucidate the precise molecular drivers behind such aggressive behavior, develop more effective and durable targeted therapies, and ultimately improve long‐term outcomes and quality of life for these exceptionally vulnerable patients. Additionally, clinicians should recognize that infant low‐grade gliomas can behave aggressively despite their benign histology and that early integration of molecular diagnostics is essential for guiding therapy and long‐term care.

## Author Contributions

**Thaer A. Tumar:** writing – original draft. **Mahdi W. Suboh:** writing – original draft, writing – review and editing. **Mones H. Atatre:** writing – original draft. **Hanin Shatrit:** supervision. **Qais M. Alnjoom:** data curation, resources.

## Funding

The authors have nothing to report.

## Consent

Written informed consent was obtained from the patient's parents for the publication of this case report.

## Conflicts of Interest

The authors declare no conflicts of interest.

## Data Availability

The data used to support the findings of this study are included in the article.
